# Polyphenol-based targeted therapy for oral submucous fibrosis

**DOI:** 10.1007/s10787-023-01212-1

**Published:** 2023-04-27

**Authors:** Chetan Hasmukh Mehta, Shivangi Paliwal, Manjunatha S. Muttigi, Raviraja N. Seetharam, Alevoor Srinivas Bharath Prasad, Yogendra Nayak, Shruthi Acharya, Usha Yogendra Nayak

**Affiliations:** 1 Department of Pharmaceutics, Manipal College of Pharmaceutical Sciences, Manipal Academy of Higher Education, Manipal, 576104 Karnataka India; 2 Manipal Centre for Biotherapeutics Research, Manipal Academy of Higher Education, Manipal, 576104 Karnataka India; 3 Department of Ageing Research, Manipal School of Life Sciences, Manipal Academy of Higher Education, Manipal, 576104 Karnataka India; 4 Department of Pharmacology, Manipal College of Pharmaceutical Sciences, Manipal Academy of Higher Education, Manipal, 576104 Karnataka India; 5 Department of Oral Medicine and Radiology, Manipal College of Dental Sciences, Manipal, Manipal Academy of Higher Education, Manipal, 576104 Karnataka India

**Keywords:** Oral submucous fibrosis, Polyphenols, Epigallocatechin 3-gallate, Quercetin, Transforming growth factor beta-1, Collagen, Fibroblasts, OSF rat model

## Abstract

Oral submucous fibrosis (OSF) is a chronic, progressive, and precancerous condition mainly caused by chewing areca nut. Currently, OSF therapy includes intralesional injection of corticosteroids with limited therapeutic success in disease management. Therefore, a combined approach of in silico*, *in vitro and in vivo drug development can be helpful. Polyphenols are relatively safer than other synthetic counterparts. We used selected polyphenols to shortlist the most suitable compound by in silico tools. Based on the in silico results, epigallocatechin-3-gallate (EGCG), quercetin (QUR), resveratrol, and curcumin had higher affinity and stability with the selected protein targets, transforming growth factor beta-1 (TGF-β1), and lysyl oxidase (LOX). The efficacy of selected polyphenols was studied in primary buccal mucosal fibroblasts followed by in vivo areca nut extract induced rat OSF model. In in vitro studies, the induced fibroblast cells were treated with EGCG and QUR. EGCG was safer at higher concentrations and more efficient in reducing TGF-β1, collagen type-1A2 and type-3A1 mRNA expression than QUR. In vivo studies confirmed that the EGCG hydrogel was efficient in improving the disease conditions compared to the standard treatment betamethasone injection with significant reduction in TGF-β1 and collagen concentrations with increase in mouth opening. EGCG can be considered as a potential, safer and efficient phytomolecule for OSF therapy and its mucoadhesive topical formulation help in the improvement of patient compliance without any side effects.

**Graphical abstract:**

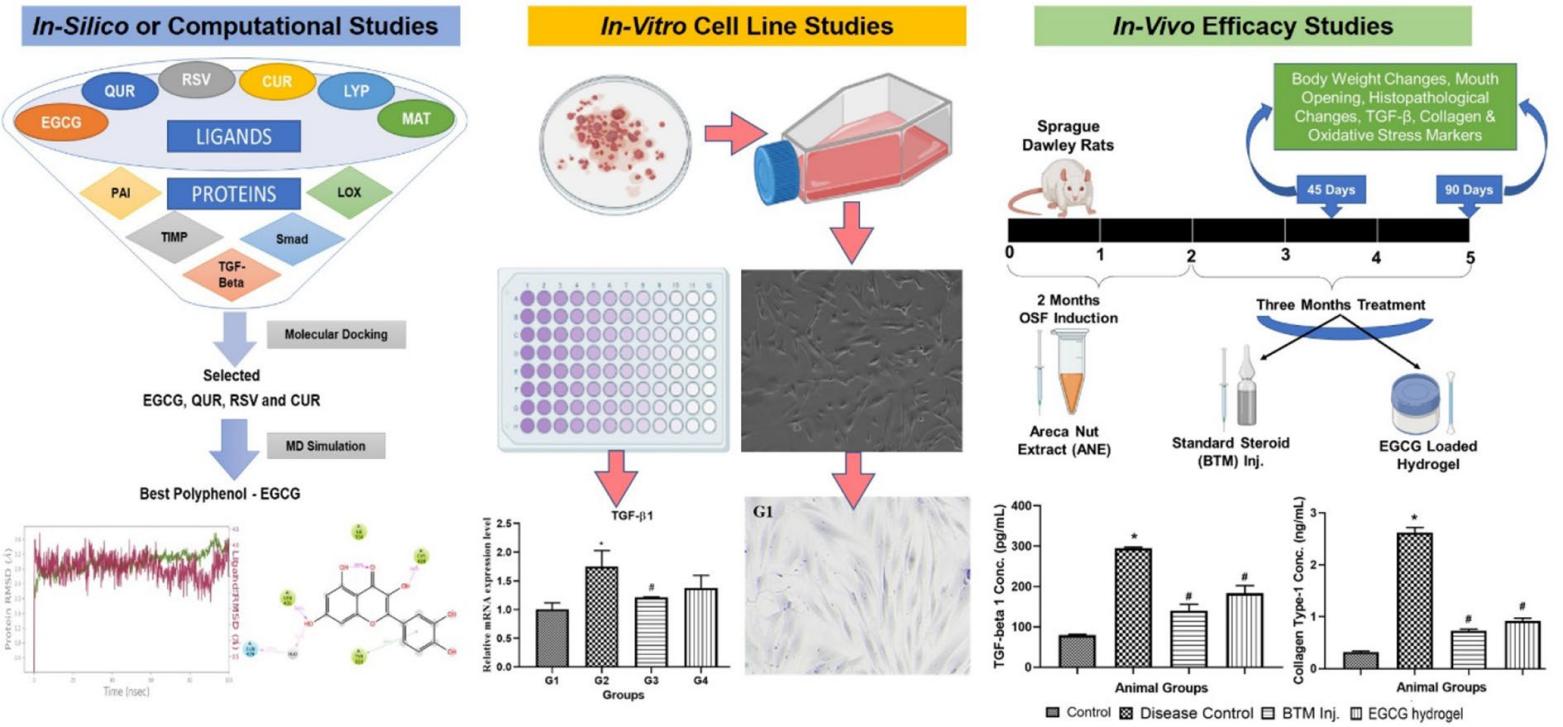

**Highlights**

- Potential polyphenols were shortlisted to treat oral submucous fibrosis (OSF) using in silico tools
- Epigallocatechin 3-gallate (EGCG) significantly reduced TGF-β1 and collagen both in vitro and in vivo
- EGCG hydrogel enhanced antioxidant defense, modulated inflammation by reducing TGF-β1 and improved mouth opening in OSF rat model.

## Introduction

Oral submucous fibrosis (OSF) is a chronic and inflammatory progressive scarring disease caused by chewing areca nut, betel quid, and gutka, resulting in the accumulation of connective tissue in lamina propria (Ray et al. 2019; Akshayak and Senthil Murugan 2021). Although this condition was observed earlier in South Asian countries, it has slowly crept into Europe and North America (Devarajan and Somasundaram 2019). It also results in serious morbidity among affected individuals. Multiple factors contribute to the disease progressions, such as defective collagen homeostasis, genetic susceptibility and immunity (Sharma et al. 2017). Arecoline is the main etiological factor responsible for OSF progression by activation of transforming growth factor-beta (TGF-β) signalling pathway. In turn, TGF-β activates other proteins, such as small mothers, against the decapentaplegic (Smad) protein, lysyl oxidase (LOX), and reactive oxygen species (ROS). These are responsible for collagen overproduction and reduction in collagen degradation by stimulating tissue inhibitors of the matrix metalloproteinase (TIMP) and plasminogen activator inhibitor (PAI) (Kondaiah  et al. 2019). Out of these proteins, TGF-β1 and LOX are the validated drug targets for inhibiting TGF-β signalling pathway (Sharma et al. 2018).

Currently, a blend of strategies have been adopted for the treatment of OSF namely habit control, drugs, surgical, physiotherapy, laser treatment and nutritional interventions. The drugs include corticosteroids (dexamethasone/ betamethasone/ hydrocortisone/ triamcinolone) alone or in combination with hyaluronidase and lycopene (LYP). The intralesional injection of corticosteroids suppresses TGF-β1 and forms the mainstay in the treatment of OSF (Shih et al. 2019). Nonetheless, none of the therapies have resulted in the successful treatment of OSF apart from rendering symptomatic relief to patients (Gopinath et al. 2022). The standard treatment, corticosteroid injection reported side effects with higher chances of infection at the site of injection and systemic side effects such as adrenal insufficiency, edema, osteonecrosis, osteoporosis, myopathies and central serous chorioretinopathy, Hence, there is a need to identify an effective strategy for the treatment of OSF, which can be an alternative to the currently available treatment and provide symptomatic relief. The literature states that non-steroidal anti-inflammatory drugs (NSAIDs), angiotensin-converting enzyme (ACE) inhibitors, and angiotensin receptor blockers (ARB) can be used to treat OSF (Wollina et al. 2015) but there efficacy is not yet proved in the treatment of OSF. As per one research studies, It was observed that among various selected patients for study, 05 (45.46%) and 06 (54.54%) patients showed mild and severe fibrosis when they were using the NSAIDs for about 6 months (Hira et al. 2016). Therefore the drugs having all the activities such as antifibrotic, anti-inflammatory and oxygen radical scavenging properties are more beneficial for the effective treatment of OSF (Xia and Li 2019; Chandran et al. 2022). The use of plant-based active moieties shows significant benefits with required safety. Medicinal plants such as cruciferous vegetables, garlic, andrographolide are explored for the treatment of different disorders such as cancer (Akkol et al. 2020; Aǧagündüz et al. 2022; Mitra et al. 2022), psychiatric and neurological disorders (Vieira et al. 2020; Akkol et al. 2020; Farooq et al. 2021), thrombotic, inflammatory and nociceptive conditions (Khan et al. 2020; Uddin Chy et al. 2021), fibromyalgia (Ferrarini et al. 2022), hyperglycemic and diabetic conditions (Mechchate et al. 2021; Haque et al. 2022), antidepressant, antidiarrheal and anxiolytic activity (Hossain et al. 2021; Jahan et al. 2022) and many other chronic disorders (Iqbal et al. 2020).

The naturally occurring polyphenols, lycopene (LYP) and curcumin (CUR) were attempted as therapeutic agents for the treatment of OSF. Saran et al. proved that polyphenol LYP is better than CUR in the management of OSF (Saran et al. 2018). Polyphenols such as epigallocatechin 3-gallate (EGCG) (Hsieh et al. 2017), quercetin (QUR) (Li et al. 2018), matrine (MAT) (Liu et al. 2018) and resveratrol (RSV) (Zeng et al. 2018) have been reported to possess antifibrotic, anti-inflammatory and oxygen radical scavenging properties. Based on the literature evidence, in the present study, an attempt was made to screen the potential polyphenols that can be used to treat OSF. Thus, in the present work, in silico studies such as molecular docking and molecular dynamic (MD) simulation were performed to analyse the molecular stability of ligands such as EGCG, CUR, RSV, QUR, LYP and MAT towards the target proteins of OSF such as TGF-β1 and LOX. The efficacy of the best polyphenols was examined using in vitro cell line studies in primary buccal mucosal fibroblasts cells and areca nut extract (ANE) induced rat OSF model.

## Materials and methods

### Materials

Epigallocatechin 3-gallate and quercetin were purchased from TCI chemical, India. Arecoline hydrobromide, ferric chloride (FeCl_3_), 2,4,6-tris(2-pyridyl)-s-triazine (TPTZ), naphthyl ethylenediamine dihydrochloride, 2,2-diphenyl-1-picryl-hydrazyl-hydrate (DPPH), Biebrich scarlet and Ponceau BS were purchased from Sigma-Aldrich-Merck, India. (3-(4,5-dimethylthiazol-2-Yl)-2,5-diphenyltetrazolium bromide), DTNB (5,5-dithio-bis-(2-nitrobenzoic acid)) also known as Elman’s Reagent, dimethyl sulfoxide (DMSO), 2-thiobarbituric acid, adrenaline bitartrate, and sodium deoxycholate were purchased from the HiMedia, India. Betamethasone injection I.P. (Betamsole, manufactured by Laborate Pharmaceuticals India Ltd) was procured from the Kasturba Hospital Pharmacy, Manipal, India. Penicillin–streptomycin (PenStrep, 5000 U/mL), GlutaMAX Supplement, Dulbecco's Modified Eagle Medium low-glucose (DMEM-Lg), Dulbecco’s phosphate-buffered saline (DPBS), and fetal bovine serum (FBS) was purchased from Gibco, Thermo Fisher Scientific, India. Weigert’s haematoxylin solution, acid fuchsine, phosphotungstic acid hydrate extrapure, phosphomolybdic acid extrapure, aniline blue (water soluble, methyl blue), sulphanilamide, molecular biology grade of ethanol, chloroform and isopropanol were purchased from the Sisco Research Laboratories Pvt. Ltd., India. Glacial acetic acid and ethanol were purchased from Merck Life Sciences Pvt. Ltd., India. RNAiso Plus, Prime Script RT reagent K (Perfect) and SYBR Premix Ex Taq (Tli RNaseH Plus) were purchased from DSS Takara Bio India Pvt. Ltd. Areca nuts extract was purchased from Vital Herbs, India. Rat transforming growth factor-beta 1 (rat TGF-β1) and rat collagen types 1 alpha 1 (rat COL1a1) enzyme-linked immunosorbent assay (ELISA) kits were purchased from Maxome Labsciences, India, and Elabscience, India, respectively. Sodium dihydrogen phosphate (NaH_2_PO_4_), disodium hydrogen phosphate, sodium carbonate (Na_2_CO_3_), potassium dihydrogen orthophosphate, disodium hydrogen orthophosphate, potassium chloride, ortho-phosphoric acid, trichloroacetic acid (TCA), butylated hydroxytoluene (BHT), hydrochloric acid (HCl), potassium dihydrogen orthophosphate (KH_2_PO_4_), disodium phosphate (Na_2_HPO_4_), hydrogen peroxide (H_2_O_2_), and sodium chloride were purchased from the S D Fine Chemicals, India. All analytical grade reagents were used.

### Methods

#### Molecular modelling

The molecular modelling platform Maestro (Schrödinger, LLC, NY, 2019) was used for performing molecular docking, followed by MD simulation studies using a workstation with Intel® Xeon® Gold 6130 Processor with 2.1 Ghz 16C/32 T 22 M cache having Nvidia Quadro P5000 graphical processing unit cards and Ubuntu 18.04.3 LTS operating system.

#### Preparation of ligands

The ligands, namely EGCG, QUR, CUR, LYP, MAT, and RSV, were taken from PubChem and drawn using a 2D sketcher in Maestro Module and converted to the 3D structure. The structures of all polyphenols were optimized using the ‘LigPrep’ tool of Schrödinger, to obtain the geometry optimized with the lowest energy at neutral pH 7.0 ± 0.0.

#### Protein preparation

The crystal structures of proteins were selected after careful screening based on their resolution. The proteins LOX (PDB ID: 5ZE3, Resolution: 2.40 Å), and TGF-β1 (PDB ID: 4X2G, Resolution: 1.51 Å) were downloaded from the ‘Protein Data Bank’ (PDB) (https://www.rcsb.org/). The structures were processed with ‘Protein Preparation Wizard’ (PPW) (Madhavi Sastry et al. 2013), wherein the missing hydrogen atoms, amino acid residues, and missing side chains were added. The proper ionization state for protein residues was generated, the water molecules beyond 5 Å were removed, and H-bond (HB) network was generated. Finally, the protein structure was minimized using the OPLS3 force field (Harder et al. 2016). A homology model was generated using 'Prime' module for the proteins for which the crystal structure was not reported (Jacobson et al. 2004). Using the BLAST tool, the protein structure with the highest homology to the query protein was selected as the template protein. Using the ClustalW, the amino acid of the query protein was aligned with the template protein. The structural model was built using the default settings in Prime. The ‘SiteMap’ tool (Halgren 2007, 2009) was used to identify the druggable, ligand-binding site for the protein.

#### Molecular docking studies

Molecular docking was performed using the ‘Glide’ module in Schrödinger to examine the molecular affinity of ligands towards the selected proteins (Friesner et al. 2004, 2006; Halgren et al. 2004). Using the Glide grid, the binding site was defined, and the ligands were docked with standard precision (SP) mode (Friesner et al. 2004, 2006).

#### MD simulations studies

The MD simulation study was performed to assess the stability between the protein and ligand using the ‘Desmond’ module in Schrödinger (Bowers et al. 2006). Initially, a solvated complex system was prepared using TIP3P water model, the system was neutralized by adding counter ions, and the iso-osmotic condition was maintained during simulation by adding 0.15 M NaCl using the ‘System Builder’ module. The solvated system was minimized using the 2000 maximum iterations and 1.0 (kcal/mol/Å) convergence threshold. The minimized system was used for running the MD simulation. The MD simulation was performed via NPT ensemble for 100 ns at 1.01 bar pressure and 300 K temperature using Nose–Hoover chain thermostat (1 ns) and Martyna–Tobias–Klein barostat (2 ns). During the simulation, 1000 structures were saved to the trajectory. The MD simulation trajectory was analyzed using the simulation interaction diagram in Desmond, and the polyphenols are graded for their best binding, and stable root mean square deviation (RMSDs).

### In vitro cytotoxicity and efficacy study

The in vitro cytotoxicity, the efficacy of EGCG and QUR were analysed using primary buccal mucosal fibroblasts cells.

#### Buccal mucosal fibroblast culture

Human buccal mucosal samples were collected after having informed consent from healthy volunteers according to the approved protocol by the Institutional Ethical Committee (IEC), Kasturba Medical College, MAHE, Manipal (#285/2021). Briefly, 4 mm biopsy specimen from third molar extraction site was taken from healthy volunteers (normal subjects with no habits of smoking and betel quid chewing of age between 18 and 32 years) using a biopsy punch and transported into a sterile medium containing 1% penicillin–streptomycin (Pen–Strep) solution (10,000 units/mL of penicillin and 10,000 µg/mL of streptomycin).

#### Primary tissue culture

The collected human buccal mucosal samples were washed three times with DPBS containing 1% PenStrep. Further, tissue samples were minced into small pieces using a sterile blade and placed on the tissue culture plate in a medium containing DMEM-Lg, 10% FBS, 1% glutaMAX and 0.5% PenStrep. Tissue culture plates were then incubated at 37 °C under 95% O_2_ and 5% CO_2_ air atmosphere (ESCO CelCulture CO_2_ Bioincubator, USA). The tissue culture plates were screened under an inverted microscope (Thermo Invitrogen EVOS M5000 Fluorescence inverted microscope, USA), and a culture medium was added every alternative day until explant monolayer cultures were 70 to 80% confluent. The fibroblasts were passaged using trypsin–EDTA and expanded in T75 flasks. Fibroblasts cryopreserved at passage 2 were used for all experiments (Adtani et al. 2018; Banerjee et al. 2021).

#### In vitro cytotoxicity studies

The effect of EGCG and QUR on viability and proliferation of primary buccal mucosal fibroblasts cells were examined using the 3-(4, 5-dimethylthiazol-2yl)-2, 5-diphenyltetrazolium bromide (MTT) colorimetric assay. Primary buccal mucosal fibroblast cells (10,000 cells per well) were seeded into each well of 96-well plate (TPP® Zellkultur and Labortechnologie, Switzerland) followed by incubation at 37 °C for 24 h in 5% CO_2_ atmosphere to allow cells to attach or adhere to the bottom of the well plate. The variable concentrations of EGCG and QUR (10, 20, 40, 80, 160 and 320 µM) were prepared in 100 µL of culture medium, added to their respective wells as per the groups, and incubated at 37 °C for 48 h in 5% CO_2_ atmosphere. After incubation, the medium was aspirated, and the cells were washed with PBS. Each well in the plate was incubated with 100 µL of MTT solution (5 mg/mL concentration) for 4 h at 37 °C in 5% CO_2_ atmosphere. After 4 h, the medium containing MTT solution was aspirated, and DMSO was added to each well to dissolve formazan crystals. The cell viability was analyzed by examining the absorbance at 570 nm and 630 nm using an ELISA plate reader (BioTek Epoch 2 Microplate Spectrophotometer, Agilent, India). All the experiments were performed in triplicates (Kamiloglu et al. 2020). The % cell viability was calculated using the following formula.

$$\% {\text{ Cell viability}} = \, \left( {{\text{Mean OD value of test sample }}/{\text{ Mean OD value of control sample}}} \right)*{1}00.$$

#### In vitro efficacy study

The in vitro efficacy of EGCG and QUR was analysed using primary buccal mucosal fibroblast cells. The pure EGCG and QUR were evaluated for mRNA expression of *TGF-β1*, *COL1A2* and *COL3A1* which are overexpressed in the OSF. The fibroblasts were seeded in the 12 well plates and 48 well plates and incubated at 37 °C for 24 h in 5% CO_2_ atmosphere. The arecoline hydrobromide solution (25 µg/mL) was supplemented in a culture medium for 48 h to induce fibrosis (Adtani et al. 2017, 2018). After confirmation of OSF induction, cells were treated with pure EGCG (10 µM) and QUR (10 µM) for 24 h and evaluated for mRNA expression of *TGF-β1*, *COL1A2* and *COL3A1* and stained for collagen deposition (Hsieh et al. 2017, 2018).

#### RNA isolation using RNAiso Plus

The media was removed from the plate, and cells were washed with ice-cold PBS. The RNAiso Plus reagent was used for cells homogenization, and cells were collected into centrifuge tubes by scrapping the plate using cell scrapper. Chloroform (molecular biology grade) was added to the above microcentrifuge tube containing cell suspension and properly mixed by vortexing vigorously. The samples were kept for 5 min at room temperature and then centrifuged at 12,000 g and 4℃ for 15 min. After centrifugation, the upper layer was separated and collected in another labelled microcentrifuge tube, followed by addition of isopropanol and mixed properly by vortexing vigorously. The vortexed samples were kept at room temperature for 10 min. and centrifuged at 12,000 g for 10 min at 4℃. The obtained RNA pellet was washed with 75% v/v ethanol by centrifugation (Sigma cooling centrifuge) at 4℃, and 7500 g for 5 min and the same procedure was repeated three times to wash the RNA pellet. The pellet was air-dried and dissolved in 20 µL of DEPC-treated water. The concentration of RNA isolated was measured using Biospectrometer (Eppendorf AG, Germany).

#### Complimentary DNA (cDNA) synthesis

The RNA was converted to cDNA by using Prime Script RT reagent K Perfect (DSS Takara Bio India Pvt. Ltd., New Delhi.) as per the manufacturer’s recommended protocol. The master mix was prepared by mixing 5 × PrimeScript buffer, PrimeScript RT enzyme mix I, oligo dT Primer (50 µM), random hexamer (100 µM), RNAase-free dH_2_O and total RNA (equivalent to 200 ng of RNA) to get total 20 µL reaction mixture. All the steps followed in dark and under cold conditions. While adding aliquots of the above-prepared mixture into a microcentrifuge tube, followed by the addition of RNA samples. The reaction mixture was incubated for three different duration and temperature conditions, first at 37 °C for 60 min for reverse transcription, second at 85 °C for 5 s for inactivation of reverse transcriptase with heat treatment, and last at 4 °C using Veriti 96 wells Thermal Cycler (Applied Biosystems, Thermo Fisher Scientific).

#### Real-time polymerase chain reaction (RT-PCR)

For performing the RT-PCR, the mixture was prepared using TB Green *Premix Ex Taq* II (2X), PCR forward primer (10 µM), PCR reverse primer (10 µM), cDNA solution and sterile purified water to get the 20 µL of the reaction mixture. The prepared samples were mixed homogeneously, and RT-PCR was performed in three steps. The first step includes initial denaturation for 30 s at 95 °C, followed by the second step, which includes conditioning of samples for 5 s and 34 s at 95 °C and 60 °C, respectively. The final steps involve dissociation by conditioning samples at three different conditions, 95 °C, 60 °C and 95 °C for 15 s, 60 s, and 15 s, respectively (QuantStudio™, Applied Biosystems, Thermo Fisher Scientific) (Adtani et al. 2018). The details of nucleotide sequences used in the study are given in Table 1.

**Table 1:** Details of nucleotide sequences used in the study

Gene	Primer Sequence (5′ → 3’)	
*TGF-β1*	Forward	ACAGCAGGGATAACACAC
	Reverse	GCAATAGTTGGTGTCCAG
*COL1A2*	Forward	GGAACTCCAGGTCAAACA
	Reverse	ACCCACACTTCCATCACT
*COL3A1*	Forward	ACTTAGAGGTGGAGCTGGT
	Reverse	TCCAAGACCTCCTCCTTTC
*18S*	Forward	GTAACCCGTTGAACCCCATT
	Reverse	CCATCCAATCGGTAGTAGCG

*TGF-β1 *Transforming growth factor beta 1, *COL1A2 *Collagen type 1A2, *COL3A1* Collagen type 3A1, *18S* house-keeping gene

#### Masson trichrome staining

After treatment duration, the Masson trichrome staining was performed on the cells using standardized protocol by Lillehei Heart Institute, University of Minnesota, USA (Adtani et al. 2018). Briefly, the media was aspirated from each well, and the cells were washed with DPBS. The cells were fixed in 4% paraformaldehyde at room temperature for 20 min. The fixed cells were washed with distilled water for 1 min. The Weigert’s hematoxylin working solution (0.2 mL) was added to each well and kept the plate at room temperature for 10 min. Further, the solution was aspirated, and the cells were rinsed with distilled water for 10 min. Biebrich scarlet-acid fuchsin solution (0.2 mL) was added to the cells and after 1 min, plate was washed quickly with distilled water. Further, a phosphomolybdic acid–phosphotungstic acid solution was added and incubated the plates at room temperature for 30 min. After aspiration of the solution, aniline blue solution (0.2 mL) was added to the plate and incubated at room temperature for 10 min. The plate was washed quickly with distilled water after aspirating the solution. The cells were treated with 1% v/v acetic acid solution for 4 min. Cells were then washed with 95% v/v alcohol followed by 100% v/v alcohol for 1 min, twice. The stained cells were observed under CKX53 microscope (OLYMPUS Medical Systems India Pvt. Ltd.).

### In vivo efficacy studies

#### Animals

The experimental protocol for in vivo efficacy and safety studies was approved by the Institutional Animal Ethical Committee (IAEC), Kasturba Medical College, MAHE, Manipal (#IAEC/KMC/112/2018). The studies were conducted as per the Committee for the Purpose of Control and Supervision of Experiments on Animals (CPCSEA) guidelines. The studies were performed in two months old male Sprague–Dawley rats weighing 200–250 g. The rats were obtained from the Central Animal Research Facilities, MAHE, Manipal. Three rats were housed per cage made up of propylene with free access to water and food. The temperature and relative humidity conditions were maintained at 25 ± 1ºC and 45–55% relative humidity with a 12 h light/dark cycle.

#### OSF induction and treatment

Sub-buccal administration of areca nut extract (ANE, 100 µL, 20 mg/mL) was given at the left buccal mucosa of rats on an alternate day for 60 days (Chiang et al. 2020). The control group of rats (*n* = 6) did not received ANE, instead water for injection was applied topically thrice a day. The disease control group (*n* = 6) received sub-buccal ANE (100 µL, 20 mg/mL) along with the buccal application of water for injection thrice a day. The third set of rats (*n* = 6) received sub-buccal ANE (100 µL, 20 mg/mL) along with once-a-week standard treatment betamethasone injection (BTM inj.). The fourth set of rats (*n* = 6) received sub-buccal ANE (100 µL, 20 mg/mL) along with thrice a day topical application of EGCG-loaded hydrogel (81.81 mg/kg). Half an hour after the application of formulation or BTM inj, rats were restricted from eating food and drinking water to avoid swallowing the formulation and to get the local mucoadhesive action of formulations on the buccal mucosa. The treatment procedure was followed for 90 days to assess the efficacy of the EGCG-loaded hydrogel (El-feky and Zayed 2019), and mucosal samples were collected from different groups of rats at the end of 90 days of treatment. During the treatment period, the rats were analysed for body weight (using a digital weighing balance) and mouth opening (using a digital Vernier caliper) once a week. After 90 days of treatment, the rats were killed, and buccal mucosa was harvested, which was used for histopathological studies by hematoxylin and eosin (H and E) and Masson trichrome method (Shekatkar et al. 2022). The concentration of collagen type-1, and TGF-β1, in the collected tissue samples, was estimated by ELISA kit (Maxome Labsciences, India, and Elabscience, India). The oxidative stress markers such as nitric oxide (NO), superoxide dismutase (SOD), catalase (CAT), thiobarbituric acid reactive substance (TBARS), 2,2-diphenyl-1-picryl-hydrazyl-hydrate (DPPH), glutathione activity and ferric reducing antioxidant power (FRAP) were analysed in tissue samples by standard methods.

#### Statistical analysis

All experimental studies were performed in triplicates, and the results were represented as mean and standard deviation. GraphPad Prism 8.0.2 was used for performing the statistical analysis. The results were analyzed according to the one-way ANOVA and considered significant if *p* < 0.05.

## Results

### Protein preparation and binding site identification

The SiteMap identified the ligand-binding pockets on the selected target protein, and based on the pocket environment analysis, the site-score and D-score were calculated for each pocket. The site-score is a measure of a particular site being the ligand-binding site, and the D-score indicates the druggability of a particular pocket (Halgren 2007, 2009). The site-score value of more than 1.0, implies a high probability that it could be a ligand-binding site. If the score is between 0.7 and 1.0, it is a partial ligand-binding site; if the score is less than 0.7, it is a non-ligand-binding site. A D-score of more than 1.0 indicates that the pocket is a druggable binding site; if it is between 0.7 and 1.0, it is a partially druggable site, and if the score is less than 0.7, it is a non-druggable site. The LOX protein was found to have three binding pockets with a site-score of 0.981, 0.975 and 1.040, while the D score for the same was 1.004, 0.945 and 1.060, respectively.

### Molecular docking studies

The ligand-binding site was unknown for LOX (PDB ID: 5ZE3) protein (Zhang et al. 2018). The SiteMap analysis identified three druggable ligand sites, site-1, site-2 and site-3. The polyphenols were docked on all three sites identified. The ligands were analyzed for intermolecular interaction with the protein individually at all the binding sites of LOX and TGF-β1. Among all polyphenols, the EGCG exhibited the highest number of combinations of polar and non-polar type of intermolecular interactions at all the binding sites of LOX and TGF-β1. The interaction pattern of all the polyphenols with LOX and TGF-β1 proteins is listed in Tables 2 and 3, respectively. Based on the interaction pattern observed across all the binding sites during molecular docking, the order of binding affinity for polyphenols towards the LOX and TGF-β1 protein is EGCG > QUR > RSV > CUR > MAT > LYP. EGCG and QUR ranked first and second for all the protein targets exhibiting higher binding interactions, whereas MAT and LYP ranked last. To further confirm the binding stability with the protein at a particular binding site, the four top-ranked polyphenols, EGCG, QUR, RSV and CUR, were subjected to the MD simulation for 100 ns.

**Table 2:** Intermolecular interaction pattern of selected polyphenols on LOX protein

Proteins	LOX site-1			LOX site-2			LOX site-3		
Ligands	HB	HP	π–π stacking	HB	HP	π–π stacking	HB	HP	π–π stacking
EGCG	GLY330 GLY331 ALA332 GLY483 SER512 SER723 SER726	ILE334 MET474 PHE484 TYR725	ARG478	ILE334 GLU336 ARG423 CYS424 THR426 GLY483	TYR333 MET429	LYS373	GLU346 ILE388 SER411 GLU555 ILE748 SER751	–	HIP747
QUR	GLY330 GLY331 ARG478 SER512 ASP724	ARG329 VAL471 MET474 TYR725	–	GLU336 CYS424 LEU431 GLN479	TYR333 ILE334 LYS373	–	ARG329 ARG338 GLU340 ILE748	ILE385 VAL551 ILE748	–
CUR	GLY330 ARG478 SER512 PRO716	TYR72 MET474 VAL713 PHE718	–	ILE334 ARG478 GLN479	TYR333	–	ARG338 ARG339 GLU340 ILE748	ILE385	–
RSV	GLY330 TYR333 GLY335 GLU336 ASP724 TYR725	ARG329 ARG478	–	GLN479	ILE334 ALA428 MET429	–	GLU340 GLU346 ALA554 GLU555	ILE385 PRO387	–
MAT	ARG478 SER723	LEU328 GLY331 ALA332 MET474 PHE484	–	THR426 ARG478 GLN479	PRO427 ALA428 MET429	–	GLU555 SER751	TRP347 PRO387 ALA554 ILE748	–
LYP	ARG329 SER723	GLY331 MET474 TYR725	–	ARG478 GLN479	PRO427 MET429 ILE334	–	ARG329 GLU340 ASN727	TRP347 PRO387 ALA554 ILE748	–

*EGCG* epigallocatechin 3-gallate, *QUR* quercetin, *CUR* curcumin, *RSV* resveratrol, *MAT* matrine, *LYP* lycopene, *LOX* lysyl oxidase, *HB* H-bonds, *HP* hydrophobic interaction, *LYS* lysine, *ASP* aspartic acid, *ASN* asparagine, *ILE* isoleucine, *VAL* valine, *SER* serine, *HIS* histidine, *GLY* glycine, *TYR* tyrosine, *ALA* alanine, *LEU* leucine, *CYS* cysteine, *MET* methionine, *TRP* tryptophan, *THR* threonine, *ASN* asparagine, *ARG* arginine, *GLU* glutamic acid

**Table 3:** Intermolecular interaction pattern of selected polyphenols on TGF-β1 protein

Ligands	HB	HP	π–π stacking
EGCG	LYS232 SER280 ASP281 HIS283 ASP290 LYS335 ASN338 ASP351	ILE211 VAL219 LEU260 LEU340	_
QUR	GLU245 TYR249 ASP281 HIS283 ASP290 ASP351	ILE211 ALA230 LEU260 LEU340	_
CUR	SER80 GLU245 HIS283 ASP290	ILE211 VAL219 LEU260 TYR282	_
RSV	GLU245 TYR249 SER280 HIS283 ASP290 ASP351	ILE211 VAL219 LEU260 LEU340	_
MAT	SER287 ASP290 LYS337	ILE211 VAL219 ALA230 GLY286 LEU340	_
LYP	TYR282 GLU284 ASP290 ARG294 LYS337	ILE211 ALA230 LEU278 PHE289	_

*EGCG* epigallocatechin 3-gallate, *QUR* quercetin, *CUR* curcumin, *RSV* resveratrol, *MAT* matrine, *LYP* lycopene, *LOX* lysyl oxidase, *HB* H-bonds, *HP* hydrophobic interaction, *LYS* lysine, *ASP* aspartic acid, *ASN* asparagine, *ILE* isoleucine, *VAL* valine, *SER* serine, *HIS* histidine, *GLY* glycine, *TYR* tyrosine, *ALA* alanine, *LEU* leucine, CYS cysteine, *MET* methionine, *TRP* tryptophan, *THR* threonine, *ASN* asparagine, *ARG* arginine, *GLU* glutamic acid

### MD simulations

**Fig. 1:**
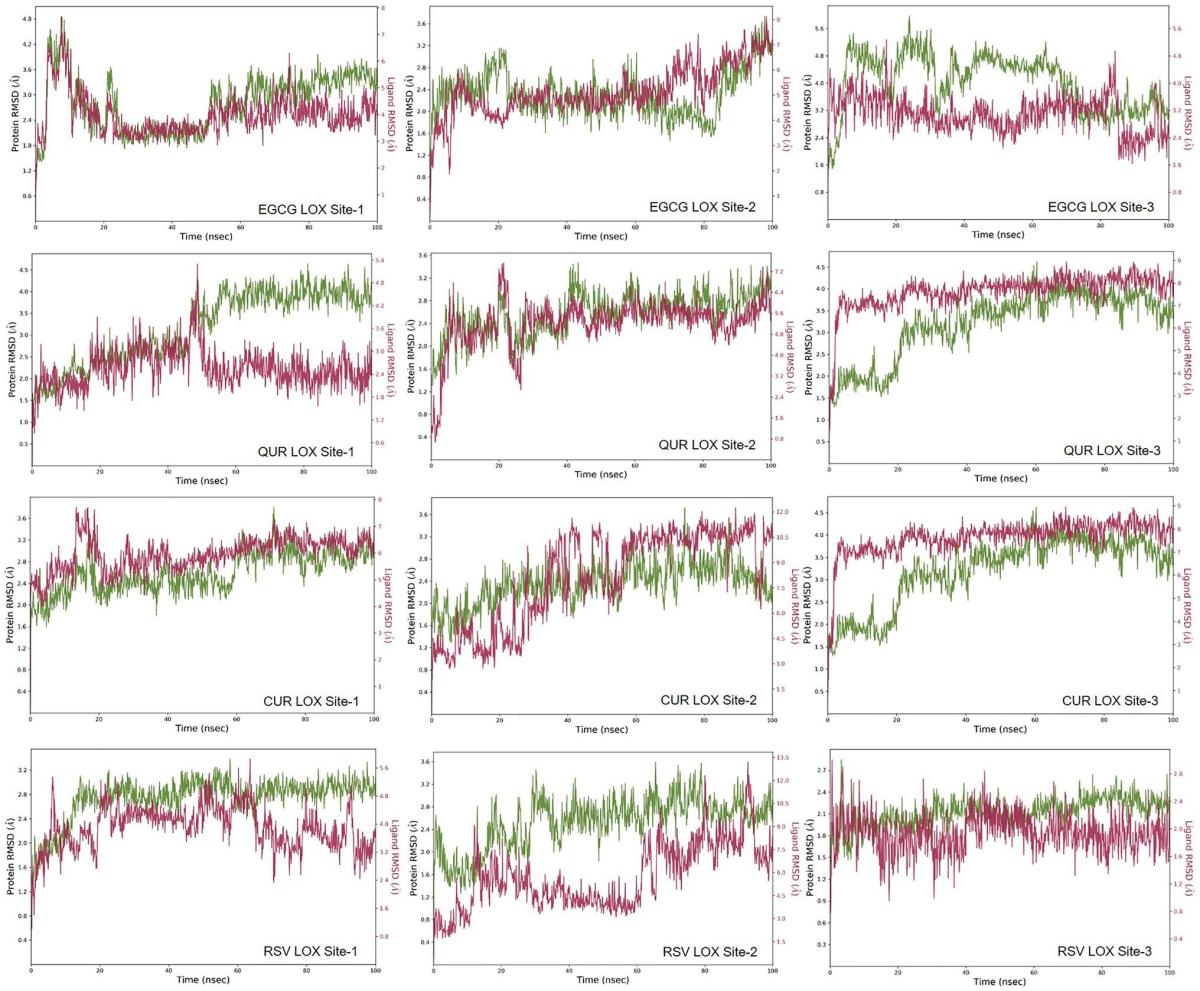
RMSD observed for the ligand (red) and the LOX protein backbone (green) during the 100 ns MD simulation. *EGCG—epigallocatechin 3-gallate, **QUR**—quercetin, **CUR**—curcumin, **RSV**—resveratrol, **MAT**—matrine, **LYP**—lycopene, **LOX**—lysyl oxidase

**Fig. 2:**
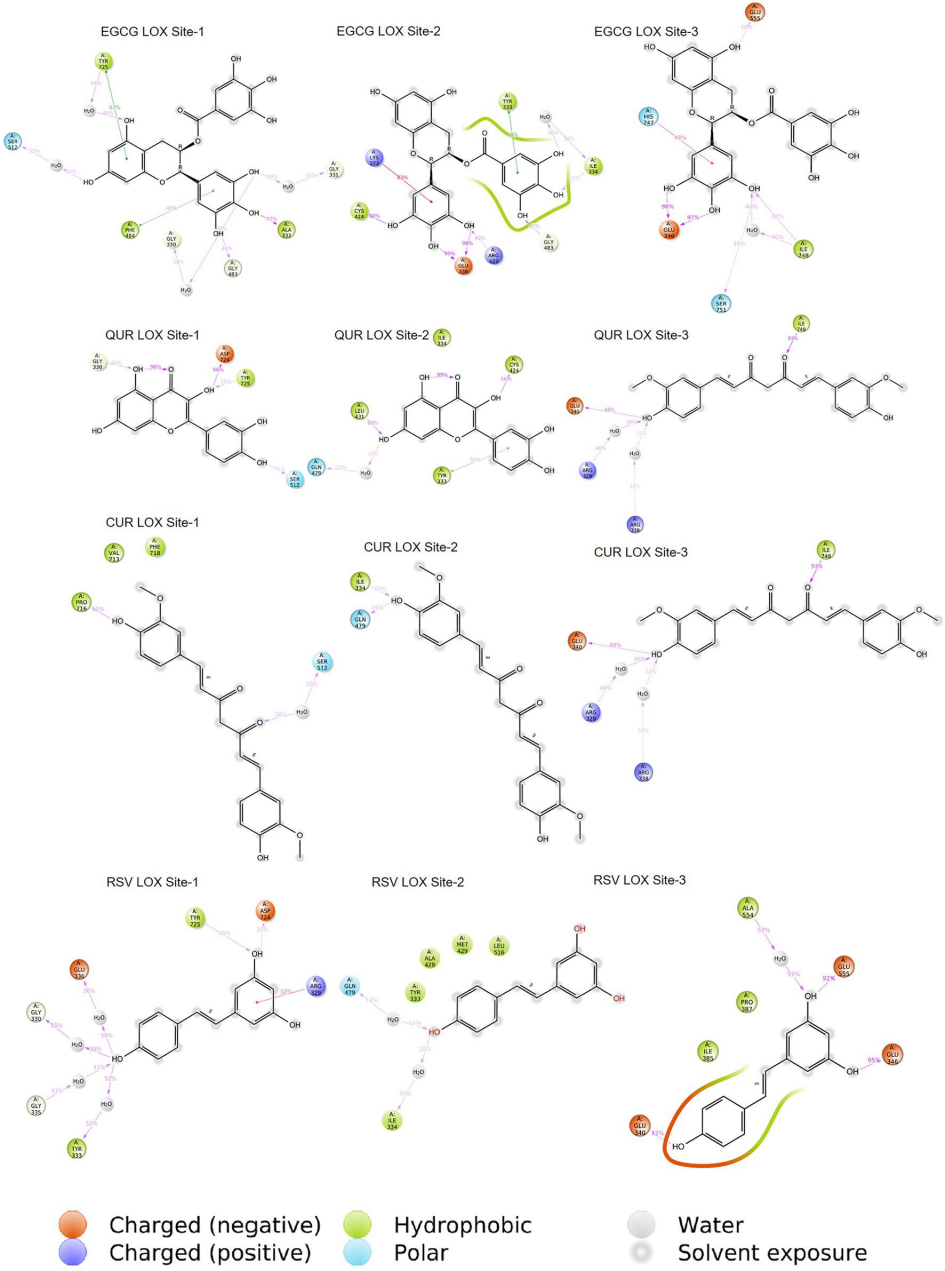
Protein–ligand contacts between LOX protein and ligands. EGCG—epigallocatechin 3-gallate, QUR—quercetin, CUR—curcumin, RSV—resveratrol, MAT—matrine, LYP—lycopene, LOX—lysyl oxidase

In the case of LOX, there were three binding sites identified in LOX, and all the polyphenols, namely, EGCG, QUR, RSV and CUR complexes with LOX at site-1, site-2, and site-3, were subjected to 100 ns MD simulation. The initial 20 ns were omitted by considering equilibration simulation for all the MD simulation runs. Except for EGCG and CUR, the RMSD fluctuations observed for the polyphenols were higher than 2 Å at the binding site-1. At binding site-2, the RMSD fluctuations were above 2 Å for all the polyphenols. EGCG exhibited higher RMSD fluctuations at the last phase of 70–100 ns simulation, whereas QUR exhibited higher fluctuations between 20 and 35 ns and the last phase of 80–100 ns simulation. At binding site-3, all the polyphenols exhibited a stable binding where the RMSD fluctuations remained at 2 Å**.** Figure 1 depicts the RMSD plot for the protein backbone and all the polyphenols and Fig. 2 illustrates the protein–ligand contacts between LOX protein and different ligands. The intermolecular interactions observed between the ligands and the different binding sites of LOX protein are listed in Table 4.

**Table 4:** Simulation interaction diagram analysis for LOX protein

Ligand protein complex	P–L RMSD	P–L Contacts (%)			Ranking
		HB	HP	WB	
EGCG LOX site-1	Stable	80.9 (ALA332) 50 (ARG478) 40 (GLY483)	30 (ILE334) 68.9 (PHE484) 80.8 (TYR725)	40 (GLY330) 30 (GLY331) 40 (SER512) 30 (SER723) 30 (SER726)	A
EGCG LOX site-2	Stable	94.6 (ILE334) 198.2 (GLU336) 40 (ARG423) 90 (CYS424) 68.9 (GLY483)	125.3 (TYR333) 84.2 (LYS373) 30 (MET429)	40 (ILE334) 50 (THR426)	A
EGCG LOX site-3	Stable	196.2 (GLU346) 40 (GLU555) 40 (SER751) 40 (ILE748)	50 (HIS747)	40 (ILE388) 40 (SER411)	A
QUR LOX site-1	Stable	45 (GLY330) 96 (ASP724)	30 (TYR725)	55 (GLY330)	B
QUR LOX site-2	Stable	68.8 (CYS424) 68 (LEU431)	68 (TYR333) 30 (ILE334)	30 (GLN479)	B
QUR LOX site-3	Stable	68.1 (GLU340) 94.7 (ILE748)	67.5 (ILE385)	65.4 (ARG329) 45 (ARG338)	A
CUR LOX site-1	Stable	55 (PRO716)	66.6 (PHE718) 40 (VAL713)	40 (SER512)	C
CUR LOX site-2	Unstable	30 (ILE334) 35 (GLN479)	50 (TYR333)	-	D
CUR LOX site-3	Stable	68.1 (GLU340) 94.7 (ILE748)	67.5 (ILE385)	65.4 (ARG329) 35 (ARG338)	A
RSV LOX site-1	Stable	35 (ASP 724) 30 (TYR725)	50 (ARG329)	50 (GLY330) 50 (TYR333) 40 (GLU336)	C
RSV LOX site-2	Unstable	–	30 (ILE334)	-	D
RSV LOX site-3	Stable	73.9 (GLU340) 95.9 (GLU346) 92.3 (GLU555)	40 (ILE385) 50 (PRO387)	50 (ALA554)	A

*EGCG* epigallocatechin 3-gallate, *QUR *quercetin, *CUR* curcumin, *RSV* resveratrol, *Mat* matrine, *LYP* Lycopene, *LOX* lysyl oxidase, *HB* H-bonds, *HP *hydrophobic interaction, *P–L* protein–ligand, *RMSD *root mean square deviation, *LYS* lysine, *ASP* aspartic acid, *ASN *asparagine, *ILE *isoleucine, *VAL* valine, *SER * serine, *HIS* histidine, *GLY* glycine, *TYR* tyrosine, *ALA* alanine, *LEU* leucine, *CYS* cysteine, *MET* methionine, *TRP* tryptophan, *THR* threonine, *ASN* asparagine, *ARG* arginine, *GLU* glutamic acid

**Fig. 3:**
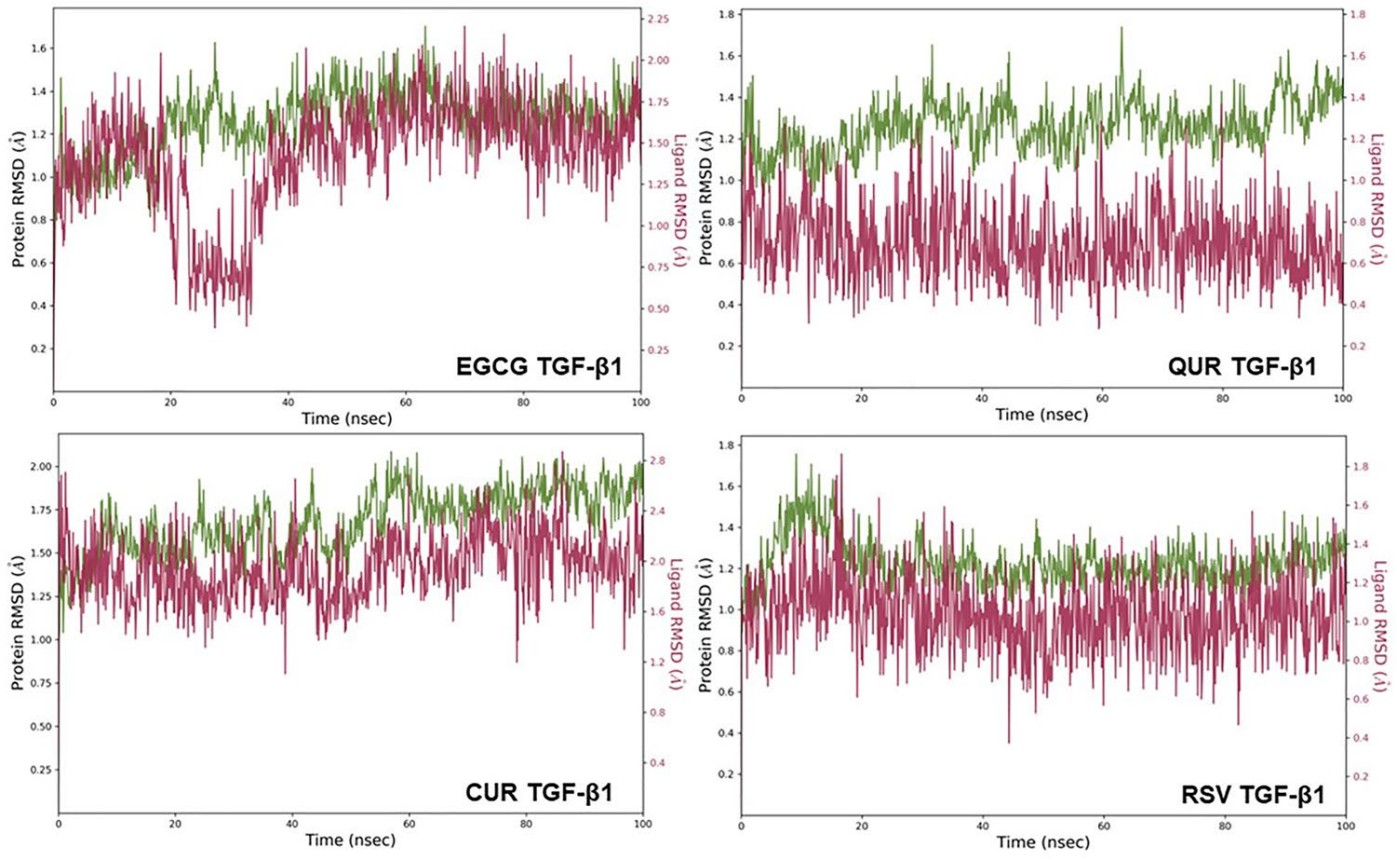
RMSD deviations observed for the ligand (red) and the TGF-β1 proteins backbone (green) during the 100 ns MD simulation; EGCG—epigallocatechin 3-gallate, QUR—quercetin, CUR—curcumin, RSV—resveratrol, TGF-β1—transforming growth factor-β1

**Fig. 4:**
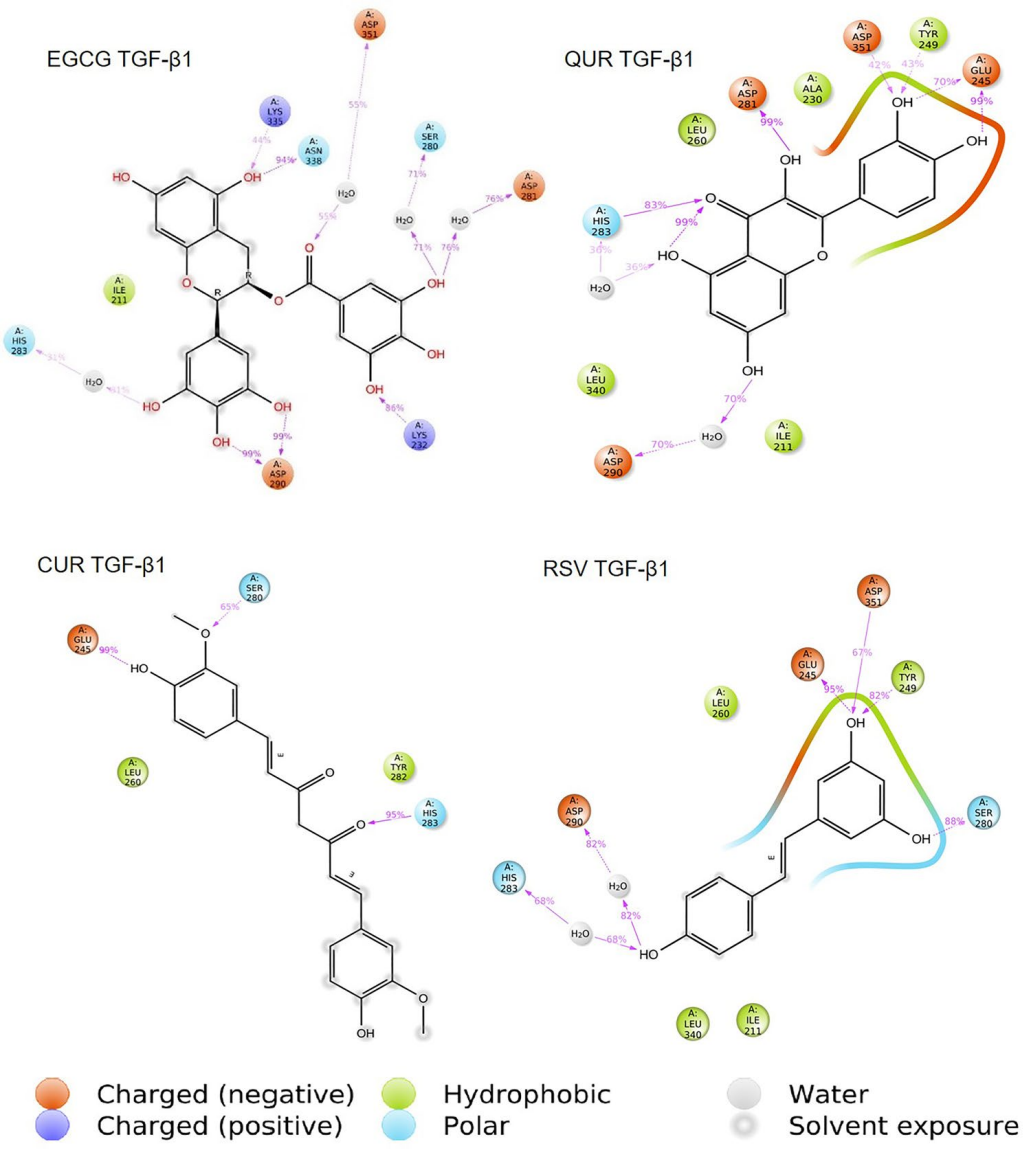
Protein–ligand contacts between TGF-β1 protein and ligands; EGCG—Epigallocatechin 3-gallate, QUR—quercetin, CUR—curcumin, RSV—resveratrol, TGF-β1—transforming growth factor- β1

EGCG, QUR, RSV, CUR complexes with TGF-β1 protein binding site were subjected to 100 ns MD simulation. The initial 20 ns simulation was omitted by considering the equilibration simulation for all the MD simulation runs. At TGF-β1 binding site, all the polyphenols under study exhibited a stable binding whereas the RMSD fluctuations remained within 2 Å. Figure 3 depicts the RMSD plot for the protein backbone and all the polyphenols, and Fig. 4 depicts the protein–ligand contacts between TGF-β1 protein and different ligands. Overall, considering TGF-β1 binding site, EGCG and RSV exhibited a stable binding compared to the polyphenols under study. The interactions observed in the trajectory frames generated during MD simulation were similar to those observed during molecular docking. The simulation interaction diagram analysis for TGF-β1 protein is shown in Table 5.

**Table 5:** Simulation interaction diagram analysis for TGF-β1 protein

Ligand protein complex	P–L RMSD	P-L Contacts (%)			Ranking
		HB	HP	WB	
EGCG TGF-β1	Stable	86.3 (LYS232) 198.3 (ASP290) 40 (LYS335) 94.7 (ASN338)	50 (ILE211) 30 (VAL219)	87.5 (SER280) 91.3 (ASP281) 50 (HIS283) 40 (ASP351)	A
QUR TGF-β1	Stable	170.7 (GLU245) 40 (TYR249) 99.8 (ASP281) 101.8 (HIS283) 71.2 (ASP351)	40 (ILE211) 60.6 (ALA230) 67.2 (LEU260) 50 (LEU340)	90.7 (ASP290)	A
CUR TGF-β1	Stable	99.3 (GLU245) 65.3 (SER280) 100.4 (HIS283)	30 (ILE211) 30 (VAL219) 55 (LEU260) 66.1 (TYR282)	40 (ASP290)	A
RSV TGF-β1	Stable	95.1 (GLU245) 82.2 (TYR249) 88.4 (SER280) 2.8 (ASP351)	30 (ILE211) 55 (LEU260) 40 (LEU340)	69.3 (HIS283) 82.9 (ASP290)	A

*EGCG* epigallocatechin 3-gallate, *QUR* quercetin, *CUR* curcumin, *RSV* resveratrol, *MAT* matrine, *LYP* lycopene, *TIMP* tissue inhibitor of the matrix metalloproteinase, *HB* H-bonds, *HP* hydrophobic interaction, *P–L* protein–Ligand, *RMSD r*oot mean square deviation. *LYS* lysine, *ASP* aspartic acid, *ASN* asparagine, *ILE *isoleucine, *VAL* valine, *SER* serine, *HIS* histidine, *GLY *glycine, *TYR *tyrosine, *ALA* alanine, *LEU* leucine, *CYS* cysteine, *MET* methionine, *TRP* tryptophan, *THR* threonine, *ASN* asparagine, *ARG* arginine, *GLU* glutamic acid

The polyphenols were scored based on binding interactions and binding stability with the target proteins based on the MD simulation studies. From the results, it was observed that all the polyphenols showed stable interactions over TGF-β1. In the case of LOX protein, EGCG interacted to a greater extent with all sites, while QUR, CUR and RSV exhibited stable interactions or binding with only site-3 as compared to site-1 and site-2. Based on these interactions, the overall scoring of the polyphenols was carried out by considering the binding of a polyphenol at the individual binding sites of each target protein. EGCG showed a higher number of interactions (4 times, 4A) as compared to the rest of the polyphenols, and therefore it was given the first rank, QUR, CUR, and RSV showed an equal number of excellent interactions (2 times, 2A), but QUR showed good interactions (2 times, 2B) as compared to CUR and RSV, thus QUR was ranked second and at last RSV and CUR showed equal better and bad interactions (1 times each, 1C and 1D) therefore they were ranked third and fourth in the list. The scoring of the selected polyphenols towards the binding site of each of the proteins is given in Table 6. The following order for the stability of polyphenols (ligands) on the selected proteins was observed: EGCG > QUR > RSV > CUR. The order of interactions indicates that EGCG demonstrated higher structural stability of the complex and the binding mode stability with all the selected proteins.

**Table 6:** Scoring of different polyphenols (ligands) based on simulation interaction diagram

Proteins	Ligands (Scoring)			
	EGCG	QUR	CUR	RSV
LOX site-1	A	B	C	C
LOX site-2	A	B	D	D
LOX site-3	A	A	A	A
TGF-β1	A	A	A	A
Total scoring	4A	2A and 2B	2A, 1C and 1D	2A, 1C and 1D

A = excellent, B = good, C = better and D = bad or no interactions

## In vitro cell line studies

### In vitro cytotoxicity studies

**Fig. 5:**
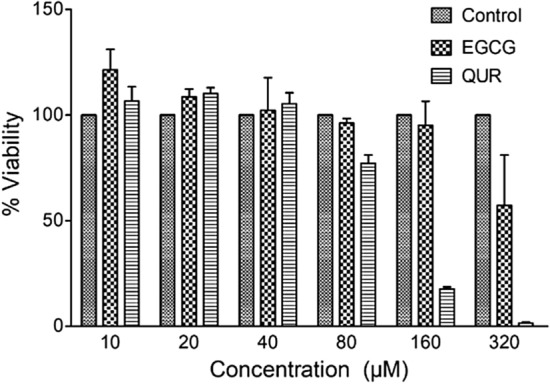
Cytotoxicity of EGCG and QUR on primary buccal mucosal fibroblasts. Values are Mean ± SD; Control: cells without treatment; EGCG: epigallocatechin 3-gallate treated; QUR: quercetin treated

The primary buccal mucosal fibroblasts cells were successfully extracted from the buccal tissue and used for analysis of EGCG and QUR efficacy on the cells. The effect of EGCG and QUR on cell viability of primary buccal mucosal fibroblast cells was evaluated using MTT colorimetric assay which suggested that EGCG and QUR at 10 µM concentration did not induce cytotoxicity in the cells whereas at 320 µM decreased cell viability (57.25 and 1.55% cell viability, respectively for EGCG and QUR). The IC_50_ values for EGCG and QUR was calculated to 342.3 and 109.1 µM, respectively. Overall, EGCG was found to be safer as compared to QUR. Based on this data, the safer concentration 10 µM was considered for further studies, including in vitro efficacy and Masson's trichrome staining studies. There was no statistically significant difference observed when different concentration groups were compared. The results of in vitro cytotoxicity at different concentrations of EGCG and QUR is shown in Fig. 5.

### Masson’s trichrome staining

**Fig. 6:**
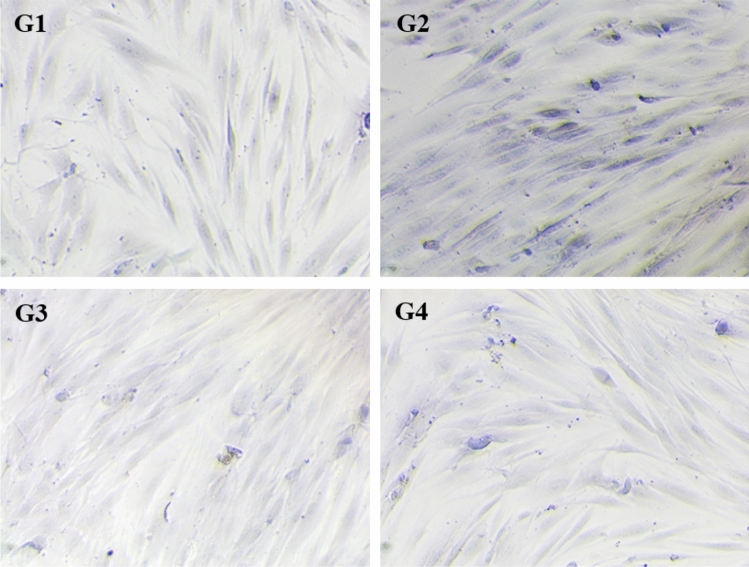
Masson's trichrome staining of primary buccal mucosal fibroblast. Comparison of collagen production qualitatively between control (G1), disease control (arecoline treated, G2) and treatment with EGCG (G3) and QUR(G4); EGCG: epigallocatechin 3-gallate; QUR: Quercetin. All the images were captured at a magnification of 100 μ (10X)

Masson's trichrome staining was performed to analyze the qualitative effect of EGCG and QUR treatment on the collagen deposition using primary buccal mucosal fibroblast cells. The qualitative effect of post-treatment with EGCG and QUR (10 µM) was evaluated on the pre-treated fibroblast cells with arecoline hydrobromide (25 µg/mL). The control group (G1) stained faintly for collagen deposition, while arecoline-treated group (G2) showed stronger staining due to higher collagen deposition. In comparison to the arecoline-treated group (G2), EGCG (G3)- and QUR (G4)-treated groups showed better improvement in collagen reduction by showing faint staining for collagen. The Masson's trichrome staining for comparing collagen production qualitatively between different groups is shown in Fig. 6.

### In vitro* efficacy studies*

**Fig. 7:**
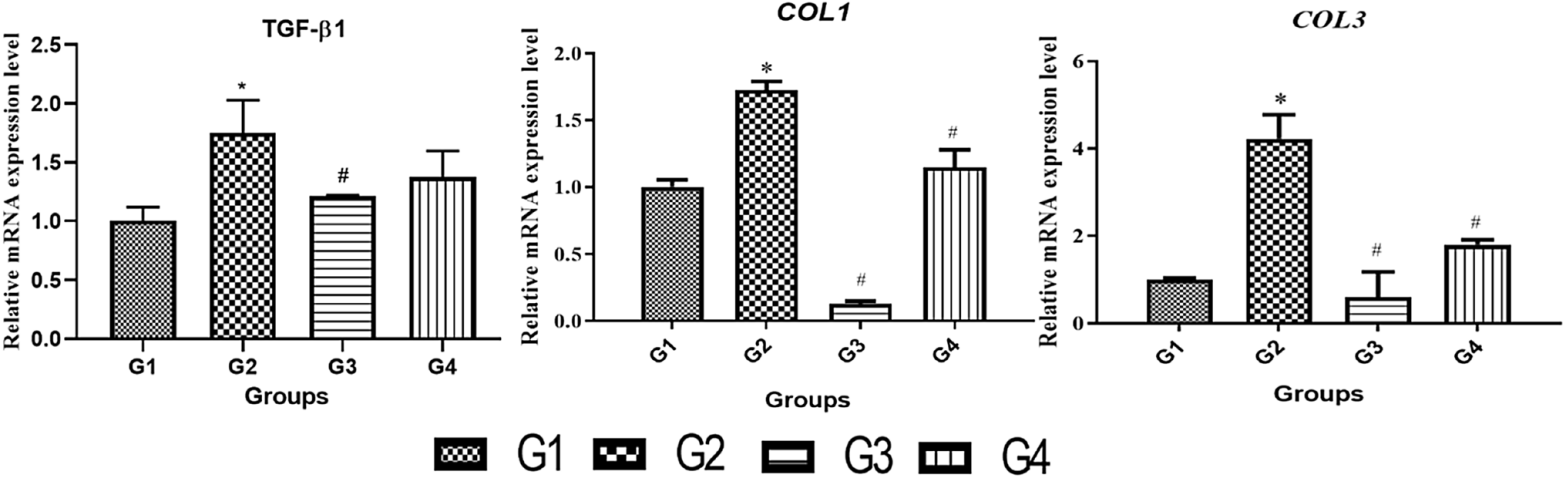
mRNA expression of *TGF-β1, COL1A2* and *COL3A1* in primary buccal mucosal fibroblasts**.** Comparison between control (G1), disease control (arecoline treated, G2), treatment groups (EGCG treated group (G3) and QUR treated group (G4); *p < 0.05 compared to G1, **#p** < 0.05 compared to G2

The effect of EGCG and QUR on the arecoline-induced OSF in vitro model was evaluated by the mRNA expression of *TGF-β1*, *COL1A2* and *COL3A1* in primary buccal mucosal fibroblast. Based on the literature, arecoline hydrobromide at 25 µg/mL was used (Adtani et al. 2018). After confirmation of fibrosis induction by 48 h, the cells were treated with 10 µM of EGCG and QUR for 24 h and after required duration, the mRNA expression of *TGF-β1*, *COL1A2* and *COL3A1* was evaluated. The mRNA expression levels of *TGF-β1, COL1A2* and *COL3A1* between different groups is shown in Fig. 7. The mRNA expression of *TGF-β1, COL1A2* and *COL3A1* significantly (*p* < 0.05) increased in the arecoline-treated group (G2) compared to the control (G1), confirming the induction of fibrosis. Treatment with 10 µM of EGCG (G3) significantly (*p* < 0.05) reduced mRNA expression of *TGF-β1, COL1A2* and *COL3A1* compared to disease control (arecoline treated group, G2). There was significant (*p* < 0.05) decrease in mRNA expression of *COL1A2* and *COL3A1 *in QUR treated group (G4) compared to the disease control group (arecoline treated, G2). EGCG showed better improvement in disease conditions by significantly reducing the mRNA expression of *TGF-β1, COL1A2* and *COL3A1*. The earlier research work performed by Hsieh and their group suggested that EGCG exhibited a promising role in inhibiting *TGF-β1*-induced collagen synthesis by suppressing early growth response-1 (EGR-1) when evaluated on human buccal mucosal fibroblasts.

### In vivo efficacy studies

**Fig. 8:**
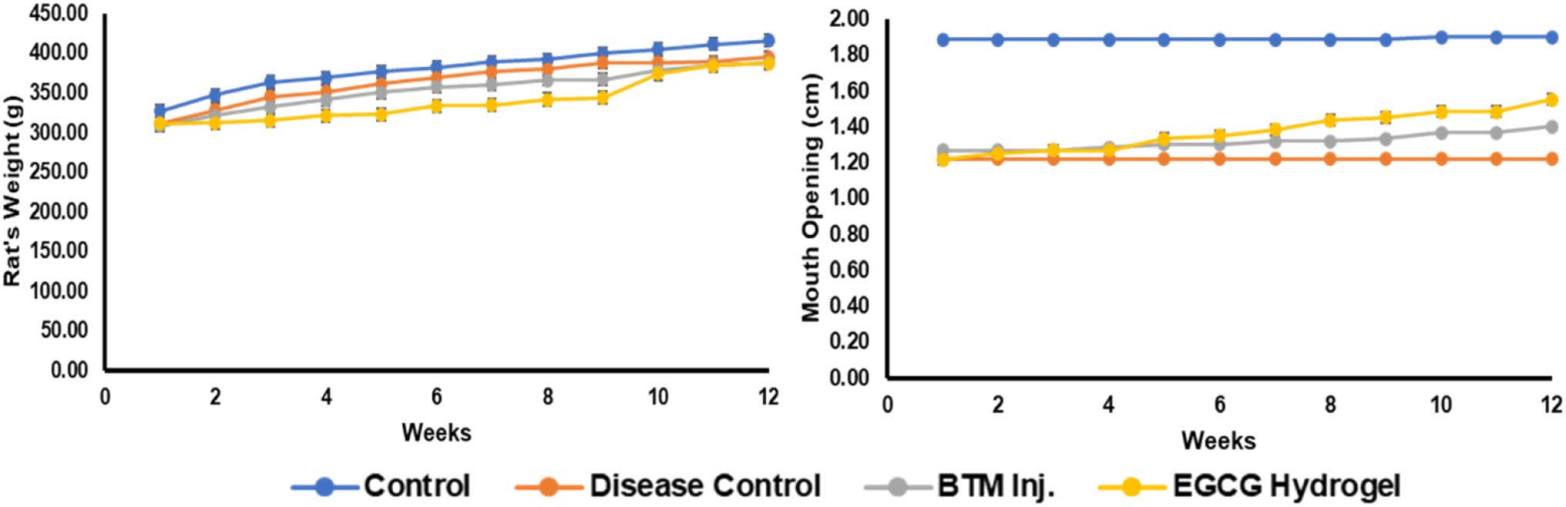
Evaluation of rat’s weight (g) and mouth opening (cm) at different time intervals (weekly), BTM Inj.—betamethasone injection

**Fig. 9:**
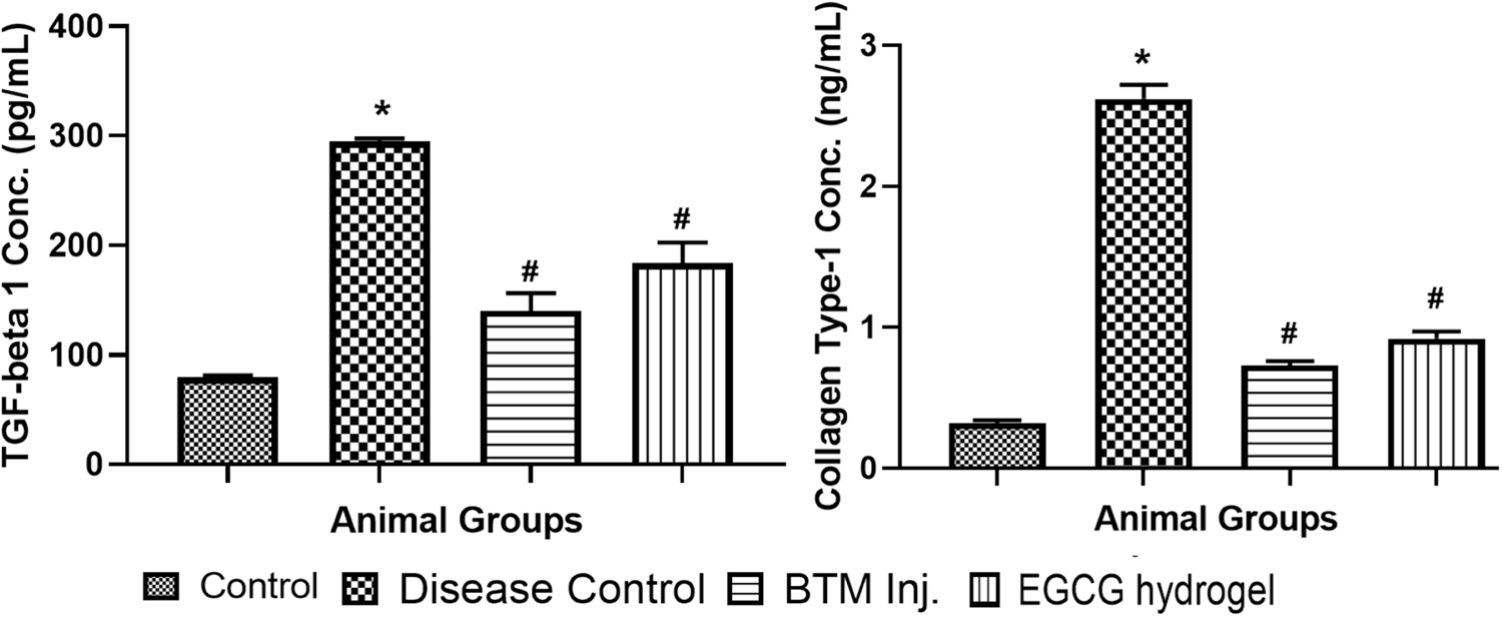
TGF-β1 and collagen-1 concentration in various animal groups. *p < 0.0001 compared to control group, #*p* < 0.0001 compared to disease control group

The efficacy of EGCG was studied in ANE induced rat OSF model. The mortality was not observed in rats during the OSF induction as well as throughout the treatment period. The changes observed in rat weight (g) and mouth opening (cm), which were evaluated every week during treatment, are shown graphically in Fig. 8. EGCG hydrogel treatment did not produce a major difference in the weight of rats, and mouth opening compared to the control rats. EGCG hydrogel treatment showed better improvement in the mouth opening compared to BTM inj. It was also notices that there were local infections in the oral cavities of BTM inj. treated rats. Induction of OSF in the rats required two months which was confirmed by a decrease in mouth opening and increase in TGF-β1 and collagen type-1 in the disease control rats. Figure 9 represents the TGF-β1 and collagen-1 concentrations in oral submucosal tissue upon treatment with BTM inj. and EGCG hydrogel. There was significant increase in TGF-β1, and collagen type-1 in the disease control group compared to the normal control group. The TGF-β1 and collagen type-1 in OSF-induced rats were significantly lower in EGCG hydrogel and BTM inj., compared to the OSF induced rats. Both EGCG hydrogel and BTM inj. showed an almost equal reduction in overexpression of TGF- β1 and collagen type-1.

**Fig. 10:**
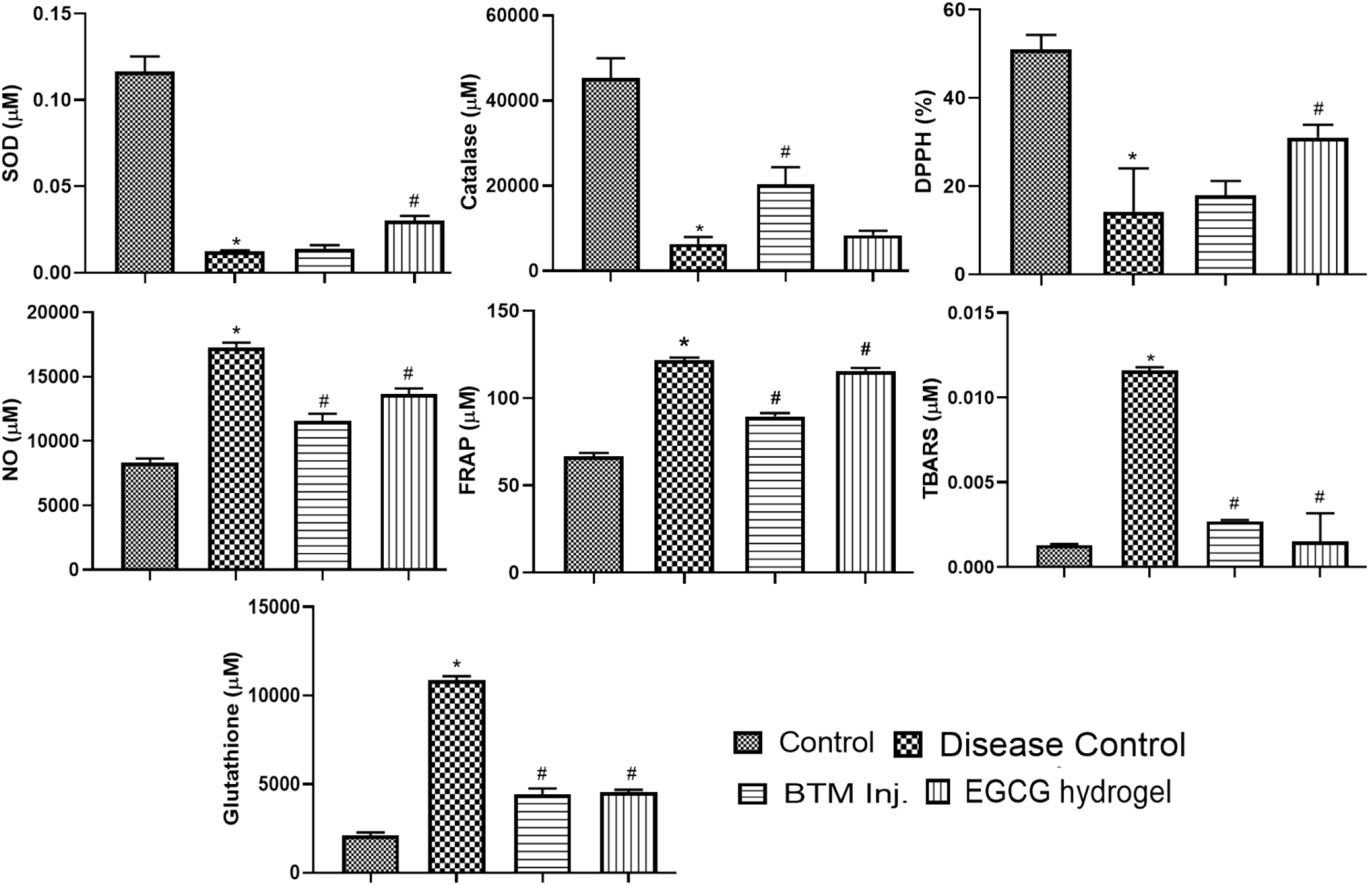
Antioxidant assays for different animal groups. *p < 0.0001 compared to control group, #*p* < 0.0001 compared to disease control group

Figure 10 represents the antioxidant parameters in the oral submucosal tissues of rats. NO, TBARS, FRAP and glutathione were found to be higher in the disease control rats compared to the normal rats, while EGCG hydrogel and BTM inj. prevented this and did not produce any significant changes compared to normal rats. The SOD, catalase. and %DPPH scavenging was found significantly decreased in the disease control rats compared with the normal control rats. There was a significant improvement in SOD and %DPPH upon treatment with EGCG hydrogel, while there was no much difference observed in BTM inj. compared to disease control rats. There was significant protection in catalase upon BTM inj.; however, the protection was not significant by treatment with EGCG hydrogel compared to disease control group.

**Fig. 11:**
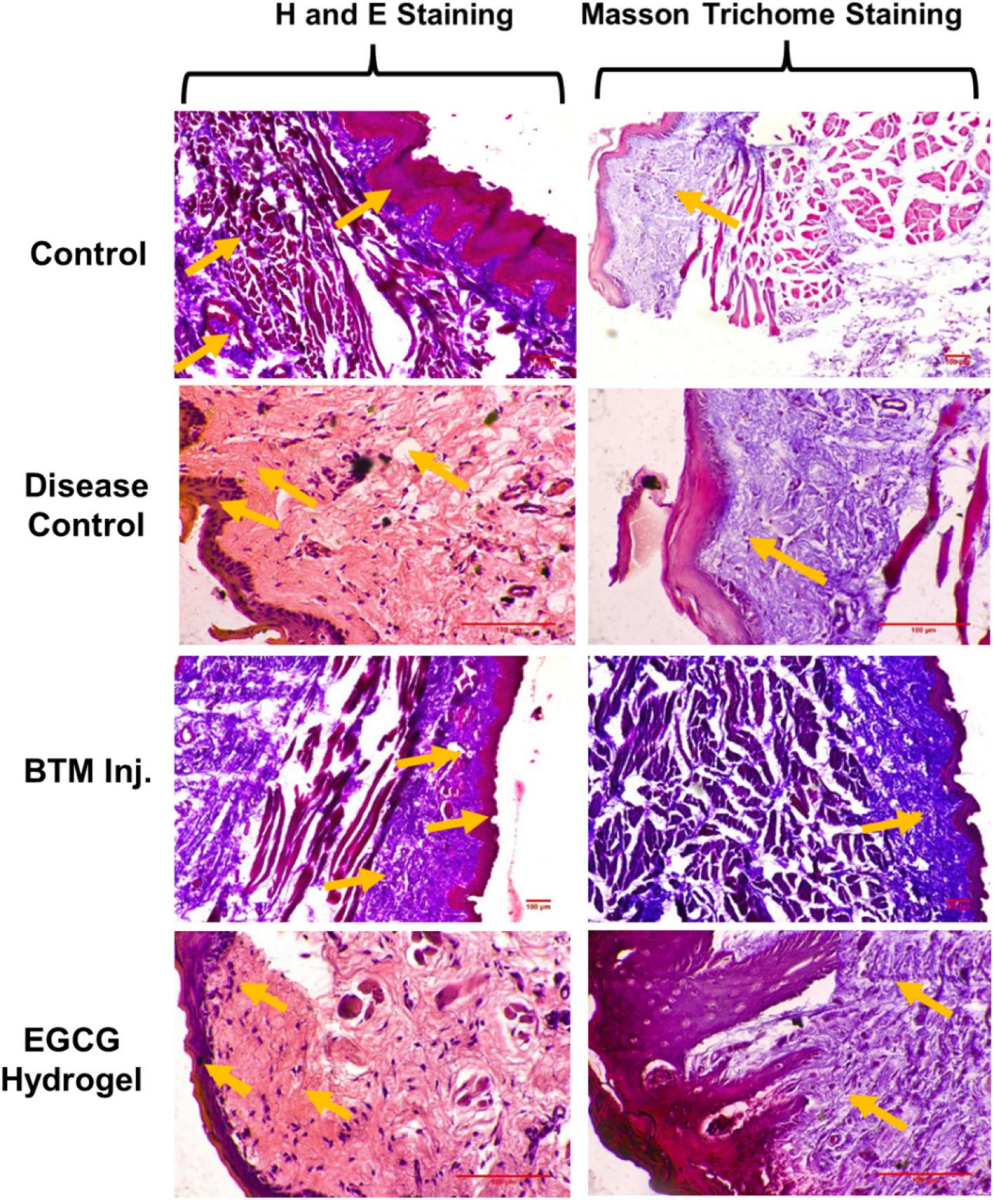
Histopathology of buccal mucosa in ANE induced OSF rats. H&E and Masson trichrome staining; The OSF was induced in rats by sub-buccal administration of ANE (100 µL, 20 mg/mL) at the left buccal mucosa of rats on an alternate day for 60 days. OSF: oral submucosa fibrosis; ANE: Areca nut extract

Figure 11 represents the histopathological evaluation by using hematoxylin and eosin (H and E) staining and Masson trichrome staining. The histology of control group showed 10–16 layers of thickness (183.83 ± 29.29 µm) stratified squamous keratinized epithelium with rete ridges. The connective tissue beneath the epithelium showed few blood vessels and muscle fibers. Whereas the disease control group showed 3–6-layered thick (44.66 ± 36.48 µm) ulcerative epithelium with the presence of scab which showed fibrin and cell debris, the large necrosed area in the dermis, granulomatous inflammation with giant cells, an abundance of chronic inflammatory cells like lymphocytes, congested blood vessels. The histopathology from rats treated with BTM inj. showed atrophic epithelium with 3–6 layers thickness (67.51 ± 11.28 µm), the connective tissue beneath the epithelium showed dense infiltration of lymphocytes, muscle fibers, and mild fibrosis areas were seen. In the histology obtained from rats treated with EGCG has stratified squamous epithelium consisting of 3–6 cell layers thickness (76.02 ± 34.76 µm) with an atrophic region. The rete ridges in the epithelium was absent and, lymphocytic infiltrates, congested blood vessels, and many macrophages were seen in the dermis. Fibrosed areas were seen in the dermis. The histology of rats in the disease control group showed the tight arrangement of collagen fibres in buccal mucosal region while normal control group showed loosely arranged collagen fibres. The BTM inj. and EGCG hydrogel treated rats showed a tight arrangement of collagen fibres in buccal mucosa.

## Discussion

The combination of in silico, in vitro and in vivo studies were performed for the selection of potential molecule and examination of its efficacy using primary buccal mucosal fibroblasts cells and ANE induces OSF rat model, respectively. To explore the nature of polypharmacology by polyphenols, in silico tools were used, and the study revealed that these polyphenols could be able to tightly bind to multiple drug targets such as TGF-β1 and LOX. The docking scores and MD simulation studies revealed, EGCG, QUR, CUR, and RSV as the top polyphenols. Further, based on the amino acid interactions, binding nature and the visual observation of ligand–protein interactions, EGCG and QUR were shortlisted for in vitro studies.

The main objective of the testing the cytotoxicity of the EGCG and QUR was to know the safer concertation of the compounds used. In the preliminary in vitro studies, EGCG showed cytotoxicity at lower concentration and QUR required higher concentration to induce the cytotoxicity on cultured human primary buccal fibroblasts. The primary buccal mucosal fibroblasts were used to see the arecoline induced cytosolic changes such as collagen deposition as detected by Masson's trichrome stain. It is the principle marker for antifibrotic activity (Adtani et al. 2019). The photomicrograms taken clearly differentiated the normal and arecoline induced fibroblastic changes. Both EGCG and QUR have prevented these changes which is evident from the microscopic analysis. Further, EGCG significantly lowered TGF-β1, but, QUR could not lower it significantly. Hence, EGCG was selected for the in vivo studies. However, both EGCG and QUR had significantly lowered mRNA expression for *COL1A2* and *COL3A1*.

The results were in according to the reported literature. Hsieh and group suggested that EGCG exhibited promising role in inhibiting TGF-β1 induced collagen synthesis by suppressing early growth response-1 (EGR-1) when evaluated on human buccal mucosal fibroblasts. They also investigated the pathways of TGF-β-induced EGR-1 expression in normal human fibroblasts and the effect of EGR-1 inhibition on the expression of *TGF-β1*. The results showed that at a concentration of 10 µM, EGCG was capable of completely attenuating the production of collagen stimulated by TGF-β1-induced EGR-1 activation in fibroblasts (Hsieh et al. 2017). They concluded that Egr-1 may be one of the key mediators of TGF-β1 stimulated fibrosis in OSF which could be a novel target for the treatment of OSF. Hence, based on this evidence, EGCG could be used as a possible agent for the prevention and treatment of OSF. Further, the same group revealed that EGCG was capable of inhibiting the activation of *TGF-β1* and connective tissue growth factor (CTGF/CCN2) induced by thrombin in a dose-dependent manner (Liao et al. 2018). However, we have proved that the binding to TGF-β1 and LOX in in silico models and in vivo proof of concept in rat model of OSF been proved. EGCG was reported for its protect human gingival fibroblasts by its anti-inflammatory properties mainly inhibiting tumour necrosis factor (TNF-α) which is the main mediator of inflammation (Karami et al. 2021). Hence, our study creates new platform for showing the efficacy of developed EGCG formulation.

The EGCG hydrogel was screened in ANE induced rat model of OSF. The areca nut chewing is the cause for oxidative stress and the low antioxidants stimulate the fibroblast to release pro-fibrotic factors to propagate in to OSF. The alkaloid arecoline present in areca nut enhances the collagen production and reduces its degradation as it activates the fibroblasts and leads to juxtaepithelial inflammatory reaction and disturbs the antioxidant levels followed by OSF generation. The ANE-induced OSF in rats is mimicking the human pathogenesis, where most of the disease markers and oxidative stress were elevated. The main markers such as mouth opening test and body weight changes were almost similar to human OSF (Maria et al. 2016; Shekatkar et al. 2022). EGCG hydrogel showed an improvement in the antioxidant level compared to the OSF-induced rats, which was due to the ability of the EGCG hydrogel to suppress the oxidants or free radicals present and improve the antioxidant activity. It also showed improvement in disease condition by significant reduction in the concentration of TGF-β1 and collagen concentration. In addition, the preparation of mucoadhesive formulation helped in the retention of EGCG at the buccal mucosal membrane and provided longer duration of action (Tran and Tran 2021; Kumar et al. 2022). Even though both EGCG hydrogel and BTM inj. showed an equal improvement in disease condition, EGCG hydrogel can be considered as more potential strategy for the treatment of OSF condition as the topical formulation can be sufficient for treatment without any side effects as the formulation contain non-toxic and plant-based active moiety. The Ayurvedic formulations such as Erandabhrishta Haritaki and Pippalyadi Choorna are reported for traditional use in oral malignancies (Chakravarthy et al. 2020). Curcumin was recently tried in patient with favorable outputs (Rajbhoj et al. 2021). The standard treatment, BTM inj. reported side effects with higher chances of infection at the site of injection, which decreases the treatment efficiency (Srikanth et al. 2017). Thus, EGCG hydrogel can be considered as safer and more effective in OSF rat model which can be further tested in human volunteers. The current study also assists in developing the novel treatment strategy for OSF therapy which can be tried clinically and fastens the recovery process of patient with reduced treatment duration.

## Conclusion

In the present study, various polyphenols were screened for the potential use in OSF therapy. The combination of in silico*, *in vitro and in vivo studies can be the best platform for selection of suitable polyphenol and examination of its efficacy and safety. EGCG and QUR showed higher affinity and stability towards the selected proteins such as TGF-β1 and LOX in in silico studies. Additionally, EGCG was effective and safer at higher concentration and helped in significant reduction mRNA expression level of *TGF-β1*, *COL1A2* and *COL3A1* in buccal fibroblast cells. Further the EGCG hydrogel had an ability and potential for reduction of TGF-β1 and collagen type-1 and mitigated the oxidative stress in OSF induced rat model. Based on these results, the polyphenol EGCG is a promising phytomolecule for OSF therapy which may improve the patient compliance.

## Data Availability

The datasets generated during and/or analysed during the current study are not publicly available due to the major part is unpublished research work in the spreadsheet, once sorted out and archived, but are available from the corresponding author on reasonable request.
